# Using Instagram to Enhance a Hematology and Oncology Teaching Module During the COVID-19 Pandemic: Cross-sectional Study

**DOI:** 10.2196/30607

**Published:** 2021-11-15

**Authors:** Julia Felicitas Leni Koenig, Judith Buentzel, Wolfram Jung, Lorenz Truemper, Rebecca Isabel Wurm-Kuczera

**Affiliations:** 1 Department of Hematology and Medical Oncology University Medical Center Goettingen Goettingen Germany

**Keywords:** COVID-19, medical education, distance learning, undergraduate medical education, digital medical education, Instagram, hematology and medical oncology

## Abstract

**Background:**

The COVID-19 pandemic necessitated the rapid expansion of novel tools for digital medical education. At our university medical center, an Instagram account was developed as a tool for medical education and used for the first time as a supplement to the hematology and medical oncology teaching module of 2020/2021.

**Objective:**

We aimed to evaluate the acceptance and role of Instagram as a novel teaching format in the education of medical students in hematology and medical oncology in the German medical curriculum.

**Methods:**

To investigate the role of Instagram in student education of hematology and medical oncology, an Instagram account was developed as a tie-in for the teaching module of 2020/21. The account was launched at the beginning of the teaching module, and 43 posts were added over the 47 days of the teaching module (at least 1 post per day). Five categories for the post content were established: (1) engagement, (2) self-awareness, (3) everyday clinical life combined with teaching aids, (4) teaching aids, and (5) scientific resources. Student interaction with the posts was measured based on overall subscription, “likes,” comments, and polls. Approval to conduct this retrospective study was obtained from the local ethics commission of the University Medical Center Goettingen.

**Results:**

Of 164 medical students, 119 (72.6%) subscribed to the Instagram account, showing high acceptance and interest in the use of Instagram for medical education. The 43 posts generated 325 interactions. The highest number of interactions was observed for the category of engagement (mean 15.17 interactions, SD 5.01), followed by self-awareness (mean 14 interactions, SD 7.79). With an average of 7.3 likes per post, overall interaction was relatively low. However, although the category of scientific resources garnered the fewest likes (mean 1.86, SD 1.81), 66% (27/41) of the student participants who answered the related Instagram poll question were interested in studies and reviews, suggesting that although likes aid the estimation of a general trend of interest, there are facets to interest that cannot be represented by likes. Interaction significantly differed between posting categories (*P*<.001, Welch analysis of variance). Comparing the first category (engagement) with categories 3 to 5 showed a significant difference (Student *t* test with the Welch correction; category 1 vs 3, *P*=.01; category 1 vs 4, *P*=.01; category 1 vs 5, *P*=.001).

**Conclusions:**

Instagram showed high acceptance among medical students participating in the hematology and oncology teaching curriculum. Students were most interested in posts on routine clinical life, self-care topics, and memory aids. More studies need to be conducted to comprehend the use of Instagram in medical education and to define the role Instagram will play in the future. Furthermore, evaluation guidelines and tools need to be developed.

## Introduction

The COVID-19 pandemic continues to have a substantial impact on medical education worldwide. Necessary governmental measures to contain the pandemic have required a significant reduction of personal contact between students, their educators, and patients and have led to the transfer of most medical teaching into the web-based realm [1]. At the University Medical Center Goettingen, continuous albeit strongly reduced bedside teaching was enabled to uphold this integral part of medical education; however, most of the teaching was conducted exclusively on the web via tutorials and livestreaming of lectures.

**Figure 1:**
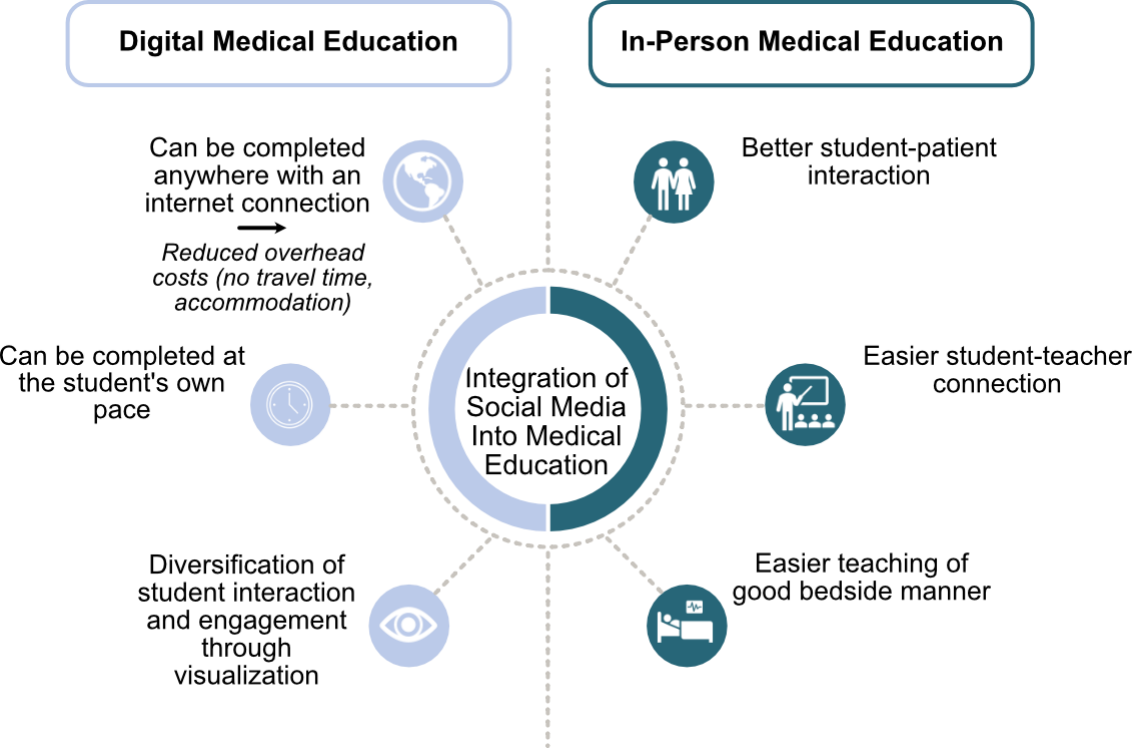
Integrating digital teaching tools: the advantages of digital versus in-person interaction.

Medical education strives to unite the teaching of scientific facts with the instruction of good bedside manner and adequate professional conduct. The relocation of medical education into the web-based realm leads to the conundrums of (1) including medical students in everyday clinical practice, (2) experiencing and learning about patient-physician interactions, and (3) establishing a sufficient student-teacher connection (Figure 1).

A recent study observed that most medical students already use smartphones and social media for education [2]. Furthermore, a growing number of clinicians utilizes social media for personal and professional purposes [3-5].

Students using social media benefit from learner engagement, customized learning, and opportunities for feedback [6]. Moreover, social media platforms allow diversification of interactions and engagements with students, as they are not limited to the written word as a medium. The COVID-19 pandemic highlighted the possibility of using social media as an effective way to deliver health care education [7].

The different social media platforms each have specific strengths and weaknesses.

**Figure 2:**
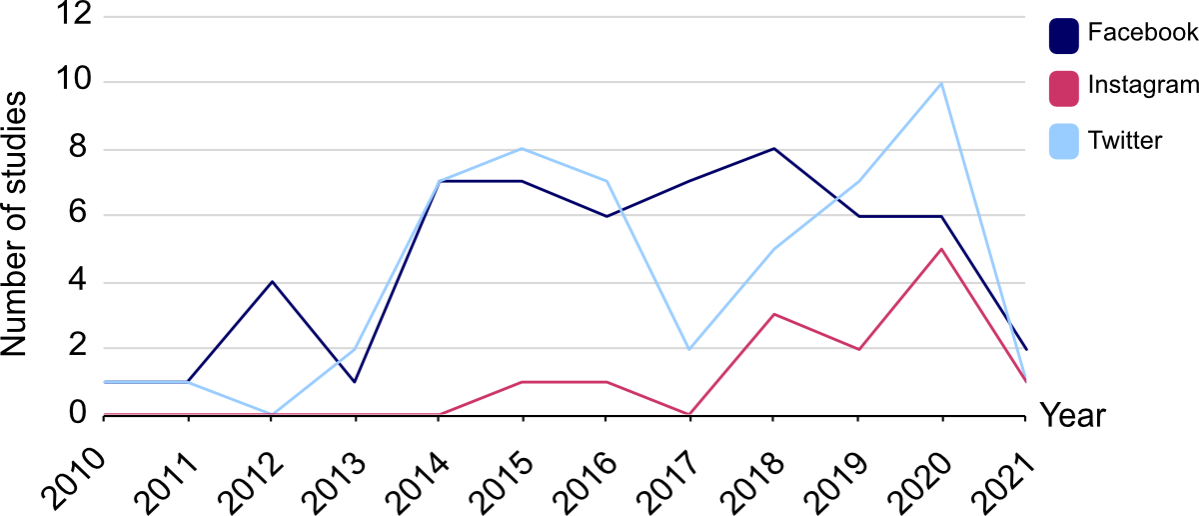
Increase in importance of social media platforms for medical education in the last decade, with a timeline of the numbers of original articles concerning specific social media platforms in the last decade.

Most research has focused on assessing the value of Twitter or Facebook as educational tools. The use of Instagram for medical education has not been extensively studied, especially in the field of hematology and medical oncology [8,9] (Figure 2). The unique selling point of Instagram lies in the strength of visualization and interaction with users, which makes the platform especially helpful for procedural training and imaging studies. To date, Instagram has been used in medical specialties such as pathology, plastic surgery, and radiology [7,8,10]. Furthermore, the platform allows insight into medical fields and physicians’ everyday routines that might otherwise only be gained during shadowing opportunities [7].

Our study aimed to explore the use of Instagram for educational purposes in the field of hematology and medical oncology to determine how to best use this platform effectively. We hypothesized that the usage of Instagram could possibly facilitate and improve interaction with medical students during this difficult time. To this end, we created an Instagram account as a tie-in for the hematology and medical oncology teaching module during the winter semester of 2020/2021.

## Methods

### Appraisal of Social Media in Medical Education

**Figure 3:**
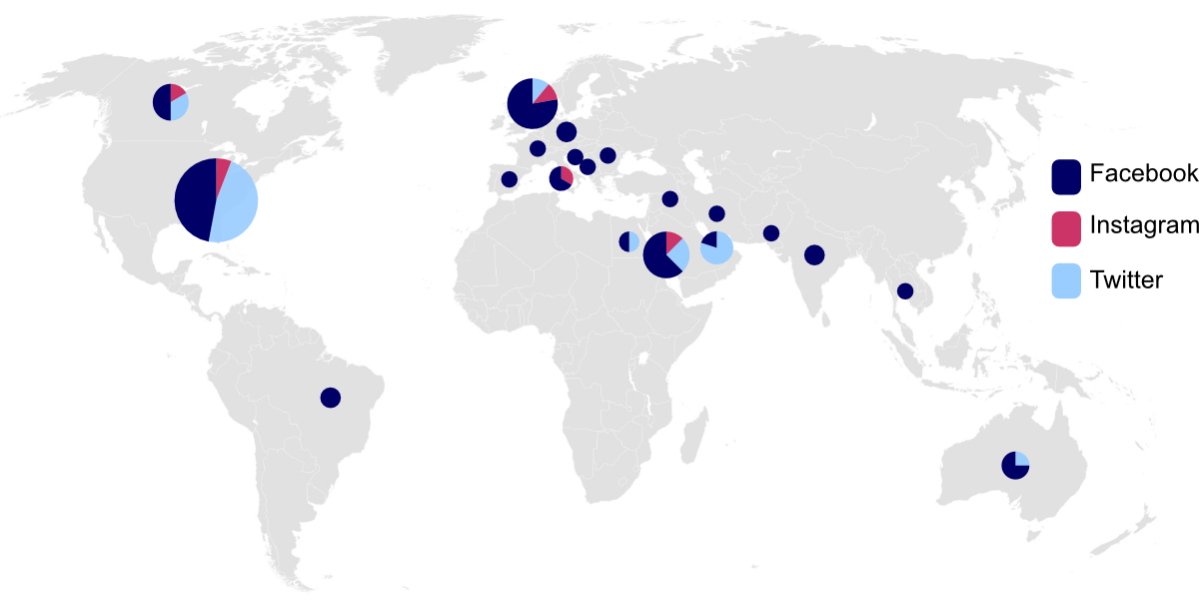
Original articles concerning the use of social media in medical education by country. The total number of original articles is represented by the circle size, and the proportion of specific social media platforms is represented by color.

An appraisal of current literature on the use of social media in medical education was conducted by searching for original articles found under the Medical Subject Headings (MeSH) terms “medical education” AND “Twitter/Facebook/Instagram” on PubMed. Studies were sorted by year of publication and country of the first author for visual analysis with Affinity Designer, version 1.9.1 (Serif Ltd) (Figure 2 and Figure 3). Only original articles investigating the use of social media in medical education were included. PubMed was chosen because there is no access restriction and the platform provides abstracts.

### Account Setup and Design

During the winter term of 2020/2021, a private Instagram account was created as a supplement to the hematology and medical oncology teaching module of the University Medical Center Goettingen. The account was only made available to medical students enrolled in this teaching module, and the students were notified about the account via their web-based class schedule and during the introductory lecture. After the students requested to follow the account, they were admitted as followers. The uploaded content on the Instagram account was synchronized to the lecture curriculum.

Uploaded content included visual comics and mnemonics, introduction to major international hematological/oncological societies, links to web-based learning resources, clinical imaging (eg, blood slides, x-ray images), guidelines, scientific papers, and personal content in which the teaching staff shared scenes of their everyday clinical practice. At least 1 post per day was uploaded, except during the holiday season, when content was uploaded once every 2 days. Each post was composed of pictures and text, which sometimes included questions. If a student left a comment via the Comments section, the teaching staff uploaded a reaction. Overall, five categories for the post content were established: (1) engagement, (2) self-awareness, (3) everyday clinical life combined with teaching aids, (4) teaching aids, and (5) scientific resources. In the engagement category, the content included an introductory message to the account for the students, holiday greetings, and good luck wishes for the upcoming examinations. The second category (self-awareness) consisted of content on resilience and self-care. The third category (everyday clinical life combined with teaching aids) included bits of everyday clinical life, such as transfusion management, blood slides, and treatment of chemotherapy-related side effects. The fourth category included clinical findings, such as hematopathology images, visual memory aids, mnemonics, and computed tomography scans. The fifth category, studies, included scientific articles (original articles and reviews); also, hematological and oncological societies were introduced (eg, the European Society for Medical Oncology and the American Society of Hematology). Approval to conduct a retrospective data analysis was obtained from the local ethics committee of the University Medical Center Goettingen (date February 25, 2021; approval 19/2/21).

### Determining Engagement

Engagement was defined as “likes” or comments and was calculated for each post separately using Excel (Microsoft Corporation) and the following formula: number of interactions per post ÷ number of followers.

### Instagram Flash Poll

After the students completed the teaching module, an Instagram poll on how the content was received was conducted. The students were informed both orally at a lecture presenting the web-based teaching module iLearn Onco and in writing via our web-based iLearn Onco script about data collection, data storage, the investigators, and the purpose of the study before the poll was opened. Only anonymous data were collected. The survey was conducted as an open survey. No incentives were offered for completing the poll. Students could answer specific questions individually, explaining the divergent number of participating students for each question. The responses were manually entered into an Excel database and analyzed. The data were collected over a time frame of 24 hours.

The poll included the following questions, which could be answered with either yes or no:

- Would you prefer funnier content?
- Would you prefer more content on the everyday clinical life of hematologists and oncologists?
- Would you prefer more mnemonics?
- Would you prefer more content on reviews and studies?
- Would you prefer more content on sensitive/personal topics?

### Statistical Analysis

Excel 2013 and GraphPad Prism, version 8.0 (GraphPad Software) were used for the statistical analysis.

## Results

### Social Media in Medical Education Is Gaining Importance

An appraisal of the current literature on the use of the social media platforms Twitter, Facebook, and Instagram for medical education showed that social media is gaining importance as an educational tool. The number of studies has slowly increased during the last decade, mirroring a shift from exclusively in-person teaching toward the integration of digital resources into medical education (Figure 2). Especially, the United States, the United Kingdom, and Arabic-speaking countries are leaders in the field, with the highest numbers of studies (Figure 3).

### Account and Follower Characteristics

During the 47 days of the hematology and medical oncology teaching module, we uploaded 43 posts accompanying our faculty’s curriculum. Of 164 students enrolled in the teaching module on hematology and medical oncology, 119 (72.6%) subscribed to the iLearn Onco Instagram account.

**Figure 4:**
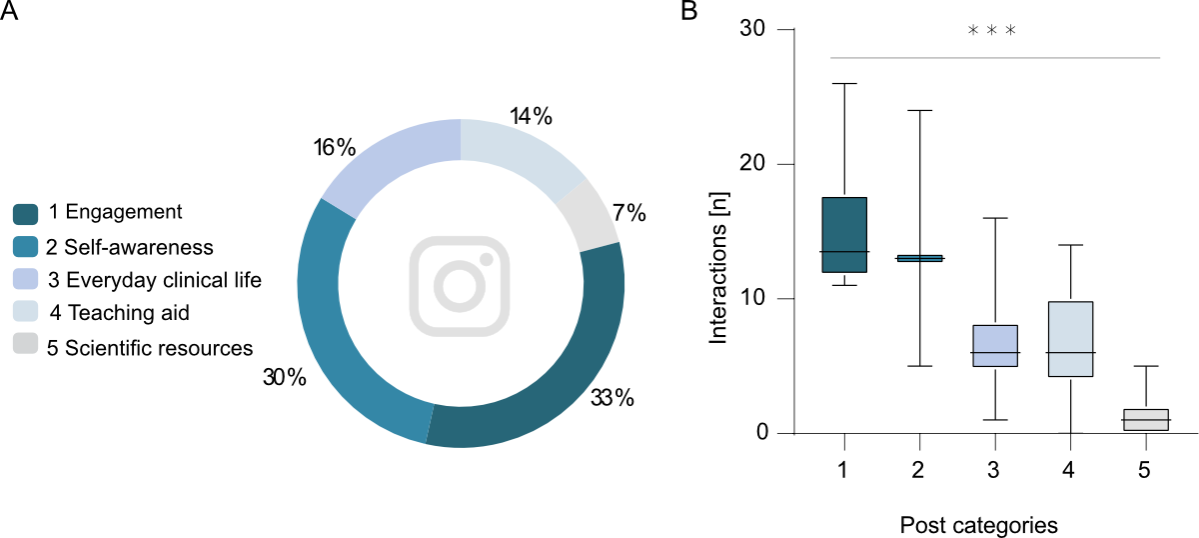
(A) Categories and proportions of content uploaded to each category. (B) Interaction of students with specific categories differs significantly (Welch analysis of variance, *P*<.01).

The first category (engagement) consisted of 6 posts, the second category (self-awareness) of 3 posts, the third category (everyday clinical life combined with teaching aids) of 14 posts, the fourth category (teaching aids) of 13 posts, and the fifth category (scientific resources) of 7 posts (Figure 4A). Of our followers, 88 (73.9%) identified themselves as female, and 31 (26.1%) identified themselves as male.

### Interaction

Our 43 posts generated 325 interactions, consisting of 315 likes and 10 comments. The mean number of likes per post was 7.33 (SD 5.71), and the mean engagement rate per post was 0.06 (SD 0.05). The most liked post had 26 likes and the highest engagement rate (0.22). Students used the comment section under the post 3 times spontaneously and answered questions asked in the comment section 7 times.

### Interaction Characteristics

Of the five different content categories established in this project, we observed the highest number of interactions for the category of engagement (mean 15.17 interactions, SD 5.01), followed by self-awareness (14.00 interactions, SD 7.79). Posts in the teaching aids category (6.85 interactions, SD 3.90) had a slightly higher interaction rate than those in the category of everyday clinical life combined with teaching aids (6.71 interactions, SD 3.53). The fewest interactions occurred with posts belonging to the category of scientific resources (1.29 interactions, SD 1.67). Interactions significantly differed between posting categories (*P*<.001, Welch analysis of variance, Figure 4B). Comparing the first category (engagement) with categories 3 to 5 showed a significant difference (Student *t* test, Welch correction; category 1 vs 3: *P*=.01, difference between means –8.45±2.45, 95% CI –14.24 to –2.67; category 1 vs 4: *P*=.01, difference between means –8.32±2.51, 95% CI –14.15 to –2.49; category 1 vs 5: *P*=.001, difference between means –13.99±2.34, 95% CI –19.63 to –8.13). There was no statistically significant difference when comparing the second (self-awareness) category to any of the other categories (Student *t* test, Welch correction; category 2 vs 1: *P*=.86, difference between means –1.17±5.95, 95% CI –21.39 to 19.06; category 2 vs 3: *P*=.32, difference between means –7.29±5.59, 95% CI –30.02 to 15.44: category 2 vs 4: *P*=.32, difference between means –7.15±5.62, 95% CI –29.62 to 15.31: category 2 vs 5: *P*=.15, difference between means –12.71±5.55, 95% CI –35.92 to 10.50). The third category (everyday clinical life combined with teaching aids) and the fourth category (teaching aids) both showed a significant difference when compared to the fifth category (scientific resources) (Student *t* test, Welch correction; category 3 vs 5: *P*<.001, difference between means –5.43±1.19, 95% CI –7.93 to –2.93; category 4 vs 5: *P*<.001, difference between means –5.56±1.32, 95% CI –8.33 to –2.79). For the third and fourth categories, compared with each other, there was no significant difference in interactions (Student *t* test, Welch correction, *P*=.93, difference of means 0.13±1.15, 95% CI –2.93 to 3.19).

### Instagram Flash Poll as a Tool for Obtaining Fast Feedback

A median of 46 (38.6%) of the 119 students who subscribed to the account participated in the voluntary Instagram poll (range 34-56). Questions could be answered individually; therefore, different numbers of participants answered each question. Of 34 students, 59% (20/34) stated they would prefer funnier content, and 66% (27/41) would have liked more information about reviews and studies. Of 56 polling students, 96% (54/56) wanted more posts about everyday clinical life, and 96% (49/51) wanted more mnemonics. All of the participating students who answered the question about sensitive/personal topics (49/49, 100%) were interested in more content on these topics. Overall, simple Instagram polls offer the opportunity for quick feedback on how content is received.

## Discussion

### Principal Findings

Although the medical community has shown increasing interest in the potential of Instagram, its role in health care has yet to be determined [8,11,12]. As a visual-based tool, Instagram offers unique possibilities for interaction. As a recent analysis illustrated [11], it can be used for patient education, patient support groups, accessibility, and medical education of either peers or medical students. We hypothesized that using Instagram as an addition to our web-based teaching curriculum could facilitate and improve interaction with our medical students during the COVID-19 pandemic. Overall, 119 (73.6%) of the 164 students in the teaching module subscribed to the Instagram account, which shows high acceptance and interest in the use of Instagram for medical education; this is in line with current data [11,12]. However, the acceptance of the account based on engagement rate, as measured in likes for specific posts, was low. Furthermore, the results of the Instagram poll were in part divergent from the number of likes, with students stating they would like more content pertaining to a category that had received few likes.

Because all students had access to the internet, either at home or provided by the faculty, and students were required to use the official web-based learning platform of the faculty, we surmise that the students who did not use Instagram actively decided against doing so.

With an average of 7.3 likes per post, interaction was relatively low [13,14]. The highest numbers of interactions were achieved for posts in the categories of engagement and self-awareness. Both categories featured content that emphasized educator and peer-to-peer connection and self-care. As this study took place during the COVID-19 pandemic, these results may reflect the current zeitgeist. A total of 3 posts addressed sensitive topics concerning the occurrence of depression and suicidal tendencies in medical students (23 likes), sleep deprivation in medical students (5 likes), and substance abuse among medical students (13 likes). These were the only contents with spontaneous positive feedback, either in the comment section or by a comparatively high number of likes. This finding may indicate that social media can be used to raise awareness for self-care and offer the possibility to advertise low-threshold services (eg, contact with the student counselor, counseling hotlines) for medical students.

The current literature on the use of Instagram in medical education and medicine in general measures interaction and engagement in likes [10]. The number of likes represents an estimate of how interesting the content is to the subscribers, and on this basis, administrators decide what content to upload [10]. In our analysis, the fifth category (studies), which contained information on current clinical trials, important reviews and studies, and web-based presentation of hematology and oncology platforms, had significantly less engagement and fewer likes than all the other categories. However, in a separate flash poll conducted on Instagram, 66% of the participating students were interested in more studies and reviews. Those findings suggest that although likes, as currently used in the literature and by industry as a measure of the interest of followers, help to estimate a general trend of interest, there are facets to interest that cannot be represented by likes [13].

Thus, to determine what types of content students are interested in, a more detailed evaluation needs to be conducted.

### Limitations

There are a few limitations to this study. First, the sample size was relatively small, and the interaction rates were low. The account was kept private, ensuring a safe space for students to learn and interact. Thus, the number of students in the study was limited by the number of students participating in the teaching module. A reason for the initially low interaction rates may be that this was the first time Instagram was used as an addition to the normally used modes of medical education at the University Medical Center Goettingen; therefore, neither students nor teaching staff were familiar with this tool as a means of education in this context. We assumed that the students had a basic knowledge of how to use and interact with Instagram, as many students already use social media in their personal lives. However, we cannot completely rule out the possibility that the decision not to use Instagram was based on a lack of training on how to use the platform.

Another limitation is that the Instagram account was created with more than one intention in mind. On the one hand, the goal was to supplement the web-based teaching module; on the other hand, we aimed to connect with students in times of social distancing and give them a feeling of inclusion in everyday clinical practice. The uploaded content that aimed to achieve each of these two goals differed greatly; therefore, the evaluation is complicated.

There is also a limitation in the scope of the review of the current literature on social media in medical education. A more comprehensive overview would require searching additional databases, such as Medline and Scopus.

### Conclusions

Instagram is a modern tool that can be easily integrated into medical teaching and allows continued personalized web-based contact between educators and students during the COVID-19 pandemic. To fully comprehend the role Instagram can play in medical education in the future, more studies need to be conducted. Furthermore, evaluation guidelines and tools need to be developed.

## Abbreviations

MeSH: Medical Subject Headings

## References

[1] Sandhu P, de Wolf M (2020). The impact of COVID-19 on the undergraduate medical curriculum. Med Educ Online.

[2] Latif MZ, Hussain I, Saeed R, Qureshi MA, Maqsood U (2019). Use of smart phones and social media in medical education: trends, advantages, challenges and barriers. Acta Inform Med.

[3] Gardner JM, McKee PH (2019). Social media use for pathologists of all ages. Arch Pathol Lab Med.

[4] Haller J, David MP, Lee NE, Shalin SC, Gardner JM (2018). Impact of pathologist involvement in sarcoma and rare tumor patient support groups on Facebook: a survey of 542 patients and family members. Arch Pathol Lab Med.

[5] Struck JP, Siegel F, Kramer MW, Tsaur I, Heidenreich A, Haferkamp A, Merseburger AS, Salem J, Borgmann H (2018). Substantial utilization of Facebook, Twitter, YouTube, and Instagram in the prostate cancer community. World J Urol.

[6] Cheston CC, Flickinger TE, Chisolm MS (2013). Social media use in medical education: a systematic review. Acad Med.

[7] Katz M, Nandi N (2021). Social media and medical education in the context of the COVID-19 pandemic: scoping review. JMIR Med Educ.

[8] Yang SC, Wu BW, Karlis V, Saghezchi S (2020). Current status of Instagram utilization by oral and maxillofacial surgery residency programs: a comparison with related dental and surgical specialties. J Oral Maxillofac Surg.

[9] Azoury SC, Mazzaferro DM, Piwnica-Worms W, Messa CA, Othman S, Stranix JT, Serletti JM, Kovach SJ, Fosnot J (2020). An update on social media in academic plastic surgery training programs: the rising trend of likes, shares, and retweets. Ann Plast Surg.

[10] Kauffman L, Weisberg EM, Eng J, Fishman EK (2020). Is a picture really worth more than a thousand words? Which Instagram post types elicit the best response for radiology education. J Digit Imaging.

[11] Wong XL, Liu RC, Sebaratnam DF (2019). Evolving role of Instagram in #medicine. Intern Med J.

[12] Gulati RR, Reid H, Gill M (2020). Instagram for peer teaching: opportunity and challenge. Educ Prim Care.

[13] De Vries ELE (2019). When more likes is not better: the consequences of high and low likes-to-followers ratios for perceived account credibility and social media marketing effectiveness. Mark Lett.

[14] de Vries L, Gensler S, Leeflang PS (2012). Popularity of Brand Posts on Brand Fan Pages: An Investigation of the Effects of Social Media Marketing. Journal of Interactive Marketing.

